# Integrating the rights-based and capability approaches in the analysis of maternal healthcare utilization in sub-Saharan Africa: A multilevel modelling study

**DOI:** 10.1371/journal.pgph.0002284

**Published:** 2023-08-29

**Authors:** Simona Simona, Nakena Likando, Andrew Banda, Million Phiri

**Affiliations:** 1 Department of Social Work and Sociology, School of Humanities and Social Sciences, University of Zambia, Lusaka, Zambia; 2 Department of Population Studies, School of Humanities and Social Sciences, University of Zambia, Lusaka, Zambia; 3 Department of Demography and Population Studies, Schools of Public Health and Social Sciences, University of the Witwatersrand, Johannesburg, South Africa; University of California San Francisco, UNITED STATES

## Abstract

The rights-based and capability approaches received increased attention relative to maternal health in the aftermath of the 2015 Millennium Development Goals (MDGs). This may be in view of the sub-optimal progress gained in reducing maternal and child mortality, especially in developing countries. Despite the combined potential of these approaches, there are limited empirical studies testing their viability in aiding our understanding of maternal healthcare utilization in developing countries. This is what this study sought to accomplish. We combined several datasets, including the Demographic Health Surveys (DHS), World Development Indicators, the World Governance Indicators and Freedom House. Bayesian multilevel logistic regression models were applied on three indicators of maternal healthcare utilization (antenatal care visits, institutional delivery, and postnatal check-ups) in relation to selected variables representing right-based and capability approaches. After controlling for relevant individual and community-level factors, the results show that living in countries with high freedom status (POR = 1.19) and higher female secondary school enrolments (POR = 1.54) increases the odds of adequate antenatal care. Residence in countries with high freedom status (POR = 1.33) and higher voice and accountability (POR = 1.72) has a positive influence on institutional delivery. Similar results are reported for postnatal care where country freedom status (POR = 1.89), voice and accountability (POR = 1.25) and female school enrolment (POR = 1.41) are significant predictors. The results imply that the rights-based and capability approaches have the potential to enhance maternal healthcare utilization in sub-Saharan Africa. Therefore, policy strategies emphasizing on freedoms, accountability, and individual capability functionings should be encouraged in the pursuit of partly achieving Sustainable Development Goals (SDG) number 3.

## 1 Introduction

The rights-based approach to health and healthcare has been the subject of much discourse since the 1948 Universal Declaration of Human Rights (UDHR). Article 25.1 of the UDHR bestows on everyone “the right to a standard of living adequate for them to enjoy good health and well-being including food, clothing, housing, medical care, social services and the right to security in the event of circumstances beyond their control such as unemployment, disability, sickness, widowhood, old age or any other lack of livelihood” [1]. Article 25.2 recognizes specifically the rights of motherhood and childhood and provides for adequate care and social protection to be given to all children regardless of how they are born. A rights-based approach to health specifically aims at realizing the right to health and other health-related human rights. Health policy making and programming are to be guided by human rights standards and principles and aim at developing capacity of duty bearers to meet their obligations and empowering rights-holders to effectively claim their health rights.

According to Leslie London [2], the rights-based approach to health is anchored on three principles and these include: 1) the indivisibility of the civil and political rights and socio-economic rights; 2) active agency of those who are vulnerable to human rights violations; and 3) the powerful normative role of human rights in establishing accountability for protections and freedoms. Civil and political rights have been well recognized in the international space over the past few decades. The rights-based approach calls for the same enthusiasm attached to civil and political rights to also apply to socio-economic rights, which includes the right to health and healthcare [2,3]. Moreover, the rights-based approach requires accountability, which combines elements of responsiveness, answerability, and redress on the part of duty bearers [4].

The rights-based approach to health gained much ground among scholars and practitioners alike post 2015 Millennium Development Goals (MDGs), especially in relation to maternal healthcare [5]. This may be due to the need for alternative approaches to maternal healthcare in view of the sub-optimal progress gained in reducing maternal mortality in the past few decades, particularly in developing countries. In the light of the sustainable development goals (SDGs) number 3, which among other things, calls for a reduction in maternal mortality ratio (MMR) of less than 70 per 100,000 live births, it is reasonable to put the rights-based approach on the centre stage of research and practice.

Generally, it is argued that access and utilization of maternal healthcare services such as antenatal care, skilled birth attendance and emergency obstetric care for all, are essential in the prevention of maternal and child mortality [6]. The insufficient progress in maternal healthcare and utilization of health services in general can partly be attributed to weak and underdeveloped health systems and lack of political commitment to improving women’s health due to their secondary status in society [5,7,8]. This means that poor political leadership contributes to inadequacies in the prevention of maternal mortality. It is within the realm of politics and public policy that national resources are distributed across the population, including health and healthcare expenditure. The rights-based framework is important in this regard as it creates the political and social benchmarks to assess the process and outcomes of development and to underscore the power hierarchies that may lead to injustices in the social, economic and health sector [9].

Furthermore, the rights-based approach provides the powerful normative role of human rights in establishing accountability for protections and freedoms of the people, especially the vulnerable [2]. It also ensures active agency by those vulnerable to human rights violations. Since national governments tend to designate deserving and undeserving claimants of rights, it is plausible to assume that this distinction depends on the extent to which governments are accountable to the population [10]. Accountability is possible only with a vigorous and well organised civil society, which is also only possible in a society that respects civil liberties and political rights [2].

A human rights approach seeks to give voice to those who are vulnerable and enable them to change their conditions for better outcomes. In this framework, rights are not only universal standards that should be followed by states, but a medium through which the suffering of people by the state, individuals acting in response to the social structure or the social structure itself, is ameliorated. Thus, individuals, groups, and communities whose rights have been or are likely to be violated should have choices and capabilities enabling them to claim their rights to better conditions [2,11–13]. In this regard, the rights-based approach is or should be complemented by the capability approach as highlighted by many proponents of both the rights-based and the capability approach [14–17].

The capability and the human rights approaches have a common motivation of fostering the dignity and freedoms of the individual. The capability approach highlights the critical importance of substantive freedoms and opportunities of individuals and groups while the human rights approach highlights the importance of values such as freedom, dignity and respect, equality and none discrimination, participation and autonomy as well as the arrangements that are needed to protect and promote these [17]. In practical terms, what people can positively achieve, including good maternal health outcomes, is influenced by several variables, including economic opportunities, political liberties, social power and the enabling conditions of good health, basic education and encouragement and cultivation of initiatives [18,19].

It is therefore, important for the rights and freedom approaches to maternal healthcare to be part of common strategies and tools to address root causes of maternal morbidity and mortality (MMM) within and beyond health systems as well as the other violations of women’s sexual and reproductive health and rights across their lives including formative gender inequalities and structural violence against women [6].

The implication of the rights-based and capability approaches is that the ‘State’ or other duty bearers commit to financial allocations necessary for making maternal healthcare services accessible and available to everyone [20]. The rights-based and capability approaches also demand opening spaces for women to exercise choices, freedoms, and subverting the social and power–relations that deny them full utilization of maternal healthcare services [4]. Additionally, the rights-based approach entails that leaders would be accountable to the population in terms of obligations to respect, protect and fulfil a wide array of civil and political rights, as well as economic and social rights, which includes health and healthcare. It is therefore expected that women who are empowered by capability functionings and live in countries that respect human rights and are accountable to the population, are better able to utilize maternal healthcare services.

However, there are still debates in literature regarding the extent to which human rights and capability functionings contribute to attainment of socioeconomic entitlements such as health and healthcare [21]. Reviewed literature indicates that there are limited studies which consider these issues especially in low resource societies like sub-Saharan Africa and many researchers call for further studies linking civil and political rights to specific outcomes [14].

The current study fills the gap in literature by examining the extent to which the rights-based and capability approaches can be integrated, and contribute to our understanding of maternal healthcare utilisation. In so doing, the study focusses specifically on the influence of country freedom status and educational attainment on maternal healthcare utilization in sub-Saharan Africa. Freedom status and education are proxies for the rights-based and capability approaches respectively. Three maternal healthcare utilization indicators are considered, including antenatal care, facility delivery, and postnatal care among women who had a live birth five years prior to the time the demographic and health survey (DHS) data was collected. Policies to bolster freedom status and reduce protracted socio-economic disadvantage experienced by women, such as inequality in education, may be what countries in sub-Saharan Africa need to improve maternal healthcare utilization. We apply Bayesian multilevel models to study the relative influence of country, community, and individual level variables on maternal healthcare utilization indicators.

## 2 Materials and methods

### 2.1 Data sources

This analysis was based on a combination of secondary and open-source datasets which are publicly available from several sources including the Demographic and Health Surveys (DHS), the World Development Indicators from the World Bank Data Bank, *Freedom in the World* report from Freedom House and the Worldwide Governance Indicators of the World Bank. The DHS is a nationally representative periodic cross-sectional survey administered to randomly selected women and men of reproductive age group in more than 90 low-and middle-income countries. The DHS follows international standards to ethically collect data on several topics including maternal healthcare. The DHS contributed 245,955 women who had given birth 5 year prior to the survey. Details on sampling, topics and ethical procedures are reported in DHS final reports and the data can be found on the DHS website: https://bit.ly/3yia3GP.

World Development Indicators (WDI) are the primary World Bank collection of development indicators, compiled from officially recognised international sources. These are the most current and accurate global development data available, and they provide national, regional, and global estimates [22]. This study used the *female secondary school enrolment* and *human development index* variables as a proxies for capability functionings at the country level and measures for each included country were derived from the WDI and Human Development Index (HDI) respectively. Freedom House is a nongovernmental organisation that has been publishing a *Freedom in the World* report on the state political rights and civil liberties of over 190 countries since the early 1970s. This dataset contributed the country-level *freedom status* variable for each of the sub-Saharan African countries. All details are found on the Freedom House website: https://freedomhouse.org.

The Worldwide Government Indicators (WGI) are research datasets summarising cross-country indicators of the quality of governance from 34 different sources capturing governance perceptions from non-governmental organisations, commercial business information providers, public sector organisations, surveys of households and firms worldwide. The WGI consist of six composite indicators of governance including voice and accountability, which was the variable included in our study representing civil liberties in the rights-based approach. They use unobserved components model statistical methodology to standardise the data from different sources to make it comparable and then aggregate weighted averages of the individual source variables to create composite indicators [23].

### 2.2 Measurements

#### 2.1.1 Outcome variables

The outcome variable for this study uses three dichotomous indicators of maternal healthcare utilization from the DHS including antenatal care visits, institutional delivery and postnatal check-ups for mothers and new-born babies. Antenatal care visits in the DHS were measured by the question which asked all women who had given birth in the last five years prior to the survey how many times they received antenatal care during the pregnancy. The responses were categorized into a binary variable with the value of ‘0’ representing at least three antenatal care visits and ‘1’ for four or more visits. Regarding institutional delivery care, the DHS asked women where they delivered their baby from for the most recent birth. Many options were given which included public hospital, private hospital, public clinic, private clinic, or home among others. The variable was recoded to either home or institutional delivery. About postnatal care, women were asked whether they and their baby(s) were checked within the first one month of delivery and their responses were also recoded into a binary variable taking the value of “0” if they were not checked and the value of “1” if they were checked.

#### 2.1.2 Country-level independent variables

The country-level independent variables include country freedom status from Freedom House and voice and accountability (VA) from the WGI. Both are used as indicators for civil and political liberties in the country, which form the core of the rights-based approach. Freedom status is derived from Freedom House’s *Freedom in the World* global report consisting of numerical ratings of political rights and civil liberties for each country. The ratings are created from 25 questions, which largely represent the UN’s UDHR [1] covering three subcategories of political rights (10 questions) and four categories of civil liberties (15 questions). Political rights questions cover electoral process, political pluralism and participation, and functioning government, while civil liberties questions include freedom of expression and belief, associational and organisational rights, rule of law, and personal autonomy and individual rights. The overall scores of both political rights and civil liberties add up 100 points. The scores are arrived at by consensus and deliberated over a series of meetings involving of more than 130 analysts, advisers, and staff with a global representation. They use a suite of data sources including newspapers, academic research, NGO reports, professional contact, and on-the-ground research. Details of the methodology can be obtained from the *Freedom in the World* methodology document [24].

The voice and accountability variable was derived from the WGI and included as an additional measure of civil liberties and political rights because it captures people’s perceptions of the extent to which the country’s citizen are able and free to engage in the selection of their government, together with freedom of expression, media freedom and freedom of association. Female secondary school enrolment variable was derived from the World Bank indicators represents the capability approach. It is measured as a percentage of gross enrolment in a country. Gross enrolment includes total enrolment, regardless of age, as a percentage of the age group that officially corresponds to the secondary level [25]. Educational status was selected here because proponents of the capability approach recognize education as a major capability functionings that give agency to individuals especially women [26]. Human Development Index (HDI) is another variable that proxies capability approach. It is a summary measure of average achievement in key dimensions of human development, including life expectancy, years of schooling and gross national income (GNI) per capita [27]. The values from each dimension are aggregated into a composite score using geometric mean. All country level variables are standardised in the analysis with the mean of 0 and standard deviation of 1 for easy comparability and interpretations. Data used here is for the 2016 iteration to be as close to the survey years of the DHS data as possible.

#### 2.1.3 Community-level independent variables

Community-level factors were aggregates of individual level variables at the primary sampling units (PSU) or cluster level because the DHS only collects individual-level data. Community level variables included community education, women’s autonomy, distance to health facilities and place of residence. These community level variables are considered as control variables for this paper, and they were selected because they are important predictors of maternal healthcare utilization as observed above. Women’s autonomy which is often used interchangeably with women empowerment or women decision-making authority for example, is selected because it signifies the agency of individual women, which an signifies individual capability. The aggregates were computed using the mean values of the proportions of women in each category of a given individual variable. Since the aggregate values may not have pragmatic meaning, the aggregate values of clusters were categorised into groups of ‘Lower’ and ‘Higher’ proportions based on national median values. Community education has three categories of ‘lower’, ‘middle’ and ‘higher’ while place of residence retains the original categorization of ‘rural’ and ‘urban’.

#### 2.1.4 Individual-level independent variables

Individual level variables included educational status, women’ autonomy, and distance to health facilities. Educational status is categorized as no education, primary, and secondary/higher. Autonomy is a composite variable from a few questions asking women about the person who usually decides on the respondent’s healthcare, large household purchases, purchases for daily use, and visits to family and relatives. In this paper, a woman who made lone decisions or together with a partner in any of the items listed was considered to have autonomy, otherwise they did not. The distance variable is derived from a question in the DHS asking residents to indicate whether they had problems with the distance to health facilities and the variable was categorized as ‘more problems’ or ‘less problem’.

### 2.3 Data management

The DHS was the main data source contributing the dependent variables and the individual and community-level data. The DHS contains standard variables across the implementing countries and for this study, data was restricted to only women who had given birth 5 years prior to the survey. All the other additional data were at the country-level and each one of these were appended to the DHS data to create a final dataset that was used for analysis. Data curation and management were done in the R computing language version 3.4.4 [28], using the data manipulation package *dplyr* [29].

### 2.4 Statistical analysis

All the Bayesian multilevel statistical models used in this study were implemented using *MLwiN* called from R through the *R2MLwiN* package [30]. In view of the large sample used in this study (245,944), our analysis did not report bivariate analysis between each of the independent variables and outcome variables. All bivariate associations were found to be significant and thus were deemed to be non-informative as the bivariate test statistic could have been affected by the large sample size. We instead reported a description of the DHS data per country including the sample size, survey year, number of clusters, median number of respondents per cluster and the range of respondents per community. We also reported results from multilevel analysis accordingly.

To determine the relative influence of contextual factors (country and community factors) on maternal healthcare utilization indicators as well examine between country and cluster variations in maternal healthcare utilization, we applied the Bayesian multilevel models. Multilevel models are appropriate because of the nature of the hierarchical data structure whereby individual women are nested within communities which in turn nested within countries. This structure violates the assumption of independence associated with standard regression analysis. Statistical models that ignore hierarchy in the data structure may underestimate standard errors and thus, make erroneous inferences [31–33].

Markov chain Monte Carlo (McMC) methods are used in the estimation of parameters. Uninformative prior distributions were specified, running for 50,000 iterations with a burn-in period of 5,000. We specifically used the Metropolis-Hastings sampling methods, which is the default algorithm for non-normal models in MLwiN. A number of tests were done to measure convergence but we retained the Raftery-Lewis diagnostics [34]. The Raftery-Lewis test shows the minimum number of iterations that would be needed to obtain the desired precision of estimation if the samples were independent. If the Raftery-Lewis diagnostics tests produced larger values than those specified in the model, the number of iterations were adjusted upwards accordingly.

We specified four models on each outcome indicator. The first model in each table will consist of an empty or null model (before the individual and community-level variables are introduced) with only contains the intercept. It is intended to measure between country and cluster variations in maternal healthcare utilization. The second model contains the country-level factors specified as country freedom status, voice and accountability, female secondary school enrolment and human development. In the third model, we introduced the community-level factors: community education, community distance, autonomy, and place of residence. The relevant individual-level control variables of educational status, female autonomy and distance were introduced in the fourth model. This approach has been used by other scholars elsewhere [35,36].

Multilevel logistic regression models are used in this study to examine the probability *p*_*ijk*_ of a woman *i* in the community *j* and country *k* having adequate maternal healthcare utilization. This analysis is represented by:

$$logit(p_{ijk})=\beta_{0}+\beta X_{ijk}+u_{jk}+v_{k}$$

where ***X***_*ijk*_ is the vector of explanatory variables at individual, community, and country levels, *ujk* is normally distributed with variance *σ*_*u*_^2^; *vk* is normally distributed with *σ*_*v*_^2^.

In terms of variances used to understand the between country and between cluster variations in maternal healthcare utilization, we used the median odds ratios (MOR) and the variance partition coefficients (VPC). The MOR is on the same scale as the odds ratios and is interpreted as the median value of the odds ratios between individuals from units at high or low risk when randomly choosing 2 individuals from different units. In this study, that would be the odds of having inadequate utilization of maternal healthcare that are determined by unexplained factors at the community and country levels.

The VPC provides information on the share of the variance at each level of analysis (individual, community, and country-levels). The VPC at each level was calculated using the latent method. It assumes a threshold model and approximating the level-1 (individual) variance by *π*^2^*/*3 (≈ 3.29) [37–40]. Higher VPC values denote that a greater share of total variation in the outcome variables is attributable to higher level membership.

$$VPCcountry=\frac{\sigma_{u(3)}^{2}}{\sigma_{u(3)}^{2}+\sigma_{u(2)}^{2}+\pi^{2}/3}$$

and

$$VPCcommunity=\frac{\sigma_{u(2)}^{2}}{\sigma_{u(3)}^{2}+\sigma_{u(2)}^{2}+\pi^{2}/3}$$

The Bayesian Deviance Information Criterion (DIC) was used to evaluate the goodness of fit of the models [41–43]. When different models are compared, a smaller DIC means the model better fits the data than one with a high DIC value.

### 2.5 Ethical consideration

The standard DHS follows international ethical standards to ensure the protection of respondents. The DHS are reviewed and approved by the ICF Institutional Review Board in the United States. The surveys are also reviewed by IRBs in all the participating countries. All the other data used in this study are publicly available and did not require any further ethical approval. More information about the DHS ethical procedures and standards can be accessed from: https://bit.ly/3P5pQAC.

## 3 Results

### 3.1 Background characteristics of participants

For this study, a total of 245,955 women (level 1) were considered, nested within 17,871 clusters or communities (level 2), within 34 sub-Saharan African countries (level 3). Table 1 presents the 34 sub-Saharan African countries included in the study, survey years, final sample per country, number of communities in a country, median number of respondents per community and range of respondents in a community. The surveys were conducted between 2006 and 2015. The total number of respondents per country ranged between the smallest, 1,445 for Sao Tome and Principe and the largest, which was 20,192 for Nigeria. The number of communities in the sample ranged from 104 for Sao Tome and Principe and 1612 for Kenya. The median number of respondents per community is between 7 and 21.

**Table 1:** Description of the DHS data by country and community (cluster).

Country	Survey year	Sample	Number of Communities	Median number of respondents per community	Range of Respondents in community
Angola	2015–16	8,947	625	15	1–26
Benin	2011–12	9,111	750	12	2–31
Burkina Faso	2010	3,960	210	17	2–49
Burundi	2010	4,916	376	13	4–21
Cameroon	2011	7,655	580	13	1–34
Chad	2014–15	11,104	626	18	3–40
Congo	2011–12	6,463	384	17	4–39
Congo DR	2013–14	11,293	540	21	9–38
Cote d’Ivoire	2011–12	5,431	352	14	4–37
Ethiopia	2011	7,764	650	13	1–26
Gabon	2012	4,143	336	12	1–34
Gambia	2013	5,385	281	17	2–72
Ghana	2014	4,294	427	9	1–33
Guinea	2012	4,999	300	16	6–41
Kenya	2014	14,949	1,612	9	1–25
Lesotho	2014	2,596	400	6	1–17
Liberia	2013	5,348	322	16	5–32
Madagascar	2008–09	8,569	600	14	4–31
Malawi	2015–16	13,448	850	16	4–27
Mali	2012–13	6,723	585	16	2–30
Mozambique	2011	7,623	611	12	2–33
Namibia	2013	3,974	600	7	1–18
Niger	2012	7,680	480	16	3–39
Nigeria	2013	20,192	904	20	3–55
Rwanda	2014–15	5,955	492	12	3–23
Sao Tome	2008–9	1,445	104	12	3–48
Senegal	2010–11	8,151	392	20	5–47
Sierra Leone	2013	8,524	435	19	5–43
Swaziland	2006–7	2,136	275	7	1–18
Tanzania	2015–16	7,050	608	11	1–18
Togo	2013–14	5,016	330	14	2–34
Uganda	2011	4,909	712	12	1–25
Zambia	2014–15	9,353	722	13	3–26
Zimbabwe	2015	4,833	400	12	2–25

**Fig 1:**
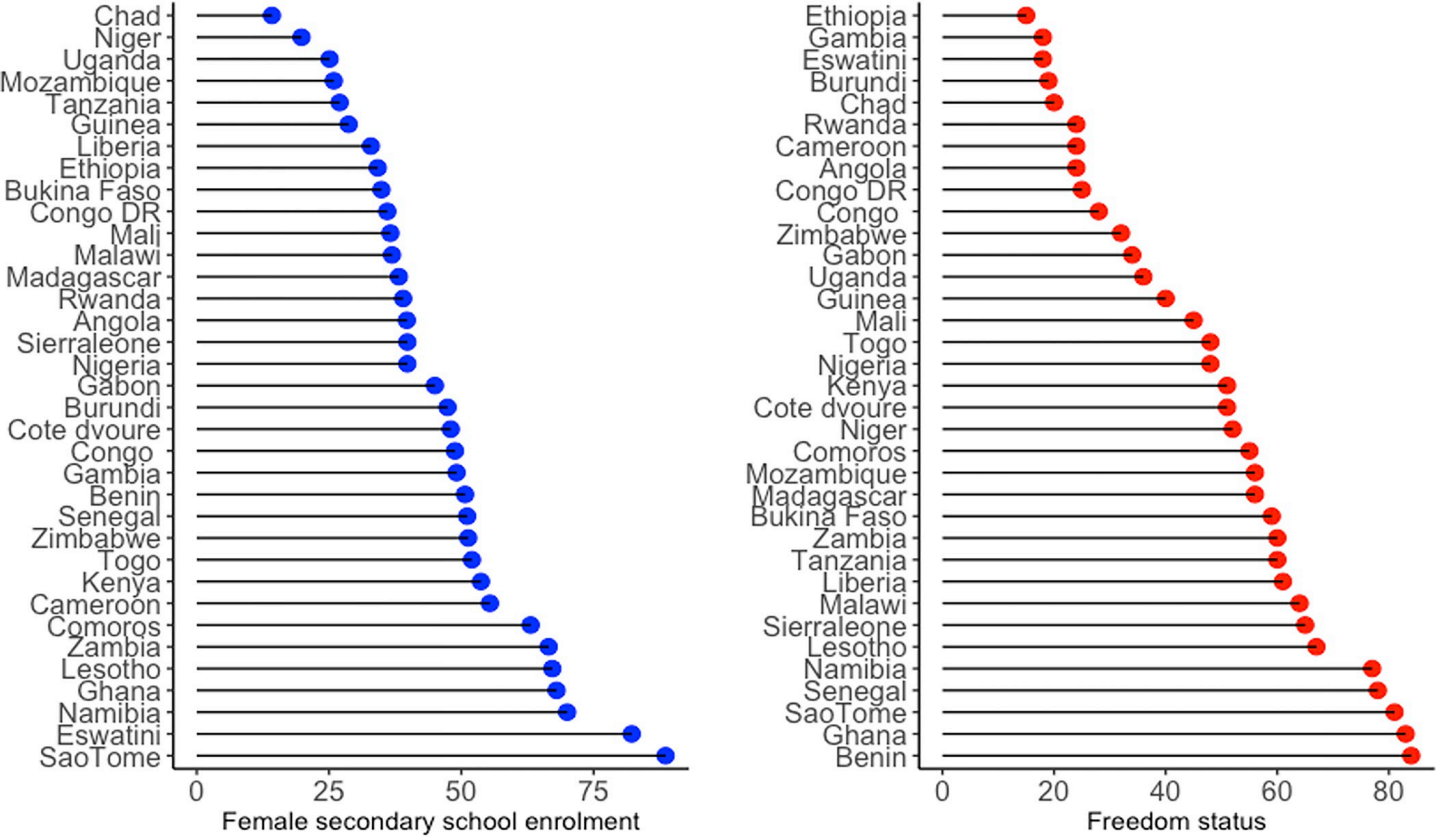
The distribution of freedom status and female secondary school enrolment.

Fig 1 reports the distribution of key independent variables across sub-Saharan Africa. It indicates that freedom status and female secondary enrolment are varied across the region. Freedom status is more pronounced in Benin, with a rating of 84, followed by Ghana at 83 and Sao Tome and Principe at 81. Ethiopia, Eswatini and The Gambia are recorded as countries where civil and political liberties are restrictive, with ratings of 15, 18, and 18 respectively. On the other hand, Sao Tome and Principe has the highest female secondary school enrolment, followed by Eswatini with Namibia in the third place. Chad is the least performing country in terms of female secondary school enrolment.

### 3.2 Freedom status, capability functionings and antenatal care in sub-Saharan Africa

A pooled Bayesian multilevel analysis was applied to understand the influence of human rights status and capability functionings on maternal healthcare utilization in sub-Saharan Africa. Four models were specified for each of the outcome variables. Table 2. reports the results of the influence of country freedom status, voice and accountability, secondary school enrolment for females, and human development index on antenatal care visits. Significant relationships are denoted by the non-inclusion of 1 in the confidence intervals and are highlighted for emphasis in these analyses. Freedom status is found to be significantly associated with antenatal care throughout the three models. For one standard deviation increase in the country freedom status, the odds of having four or more antenatal care visits increases by a factor of 1.01–1.30 after relevant factors are controlled for.

**Table 2:** Posterior odds ratios for multilevel logistic regression for freedom status, capability functionings and antenatal care in sub-Saharan Africa with 95% credible intervals (N = 245,955).

Variable	Model 1	Model 2	Model 3	Model 4
**Country-level variables**				
Intercept	1.45(1.30,1.62)	0.71(0.63,0.80)	0.90(0.71,1.06)	0.47(0.43,0.52)
Freedom status		**1.12(0.97,1.23)**	**1.23(1.10,1.33)**	**1.19(1.01,1.30)**
Female secondary school enrolment		**1.43(1.29,1.56)**	**1.60(1.37,2.05)**	**1.54(1.31,1.72)**
voice and accountability		**0.49(0.43,0.56)**	1.00(0.82,1.21)	1.09(0.92,1.41)
Human development		**1.31(1.15,1.51)**	**0.79(0.72,0.93)**	0.92(0.70,1.06)
**Community controls**				
Community education				
Low			1	1
Medium			**2.10(1.99,2.21)**	**1.60(1.52,1.67)**
High			**3.35(3.08,3.44)**	**1.84(1.73,1.98)**
**Community distance problem**				
Low			1	1
High			**0.78(0.75,0.81)**	**0.88(0.85,0.93)**
**Community autonomy**				
Low			1	1
High			**1.23(1.19,1.28)**	**1.17(1.14,1.19)**
**Residence**				
Urban			1	1
Rural			**0.63(0.61,0.66)**	**0.86(0.82,0.90)**
**Individual controls**				
Educational status				
No education				1
Primary				**1.35(1.31,1.38)**
Secondary/higher				**1.87(1.81,1.95)**
**Distance**				
Less problems				1
More problems				**0.87(0.85,0.90)**
**Autonomy**				
No				1
Yes				**1.17(1.14,1.19)**
**Random effects**				
**Country-level**				
Variance (SE)	0.76(0.19)	0.44(0.12)	0.42(0.12)	0.43(0.12)
VPC* (%)	15.2	9.32	9.61	9.91
MOR*	2.3	1.88	1.86	1.87
**Community-level**				
Variance (SE)	0.95(0.057)	0.99(0.02)	0.66(0.01)	0.62(0.01)
VPC (%)	19.00	20.97	15.10	14.20
MOR	2.53	2.58	2.17	2.12
DIC*	288,168.99	270,625.48	269,179.56	229,089.90

Female secondary education is another factor of great importance to antenatal care. It was found that living in countries with higher secondary school female enrolment increased the propensity of antenatal care by a factor of 1.31–1.72. This is after the relevant variables are taken into consideration. Education has always been an important predictor of antenatal care at the community level [44]. This finding introduces an important dimension because unlike other common studies, education is also measured at the country-level. Voice and accountability seem to only be significantly associated with antenatal care only after community and individual level variables are controlled for. The opposite is the case with human development index which is only associated with antenatal care before individual and community level variables.

In terms of control variables at the community level, education, distance to health facilities and autonomy and place of residence are significantly associated with antenatal care. The results show that living in communities with a high proportion of women with secondary or higher education, who are autonomous and have less problems with distance to health facilities increases the odds of having adequate antenatal care visits. All individual level variables are also significantly associated with adequate antenatal care visits.

The analysis also examined between community and between country variations in antenatal care visits. VPCs and MORs were calculated for all models for this purpose. Individual-level factors seem to have a bigger share in determining antenatal care visits compared to contextual factors–community and country-level. The results indicate that 15.2% and 19.0% of variance in antenatal care is explained by country and community-level factors respectively. The VPCs are significantly large which indicates the importance of community and country-level factors in explaining the cross-country variations in antenatal care visits in sub-Saharan Africa. MORs results also bolster the importance of contextual factors because they are way above 1 in all the models, indicating the large influence of higher-level factors on antenatal care. It is interesting to note that the significant drop in the VPC values when country and community-level factors are introduced. The DIC values show that they are decreasing with additional variables which implies that the full model is a better fit that the other models.

### 3.3 Freedom status, capability functionings, and institutional delivery in SSA

The influence of country freedom status and capability functionings on institutional delivery was estimated using multilevel models and reported in Table 3. Country freedom status is again showing to be an important predictor of maternal healthcare utilization. Country freedom status is associated with institutional delivery in the sense that women living in countries with higher freedom scores are more likely to deliver in health facilities. For a one standard deviation increase in the country’s freedom status, the odds of delivering in a health facility are expected to increase by a factor of 1.17–1.53 after controlling for relevant variables.

**Table 3:** Posterior odds ratios for multilevel logistic regression for freedom status, capability functionings, and institutional delivery in sub-Saharan Africa with 95% credible intervals (N = 245,955).

Variable	Model 1	Model 2	Model 3	Model 4
**Country-level variables**				
Intercept	**2.41(2.09,3.03)**	**1.89(1.60,2.34)**	**3.21(2.66,3.91)**	**1.83(1.60,2.15)**
Freedom status		1.38(0.19,1.52)	**1.29(1.11,1.41)**	**1.33(1.17,1.53)**
Female secondary school enrolment		**1.57(1.238,1.79)**	1.22(0.99,1.41)	1.19(0.90,1.62)
Voice and accountability		**0.73(0.58,0.95)**	1.27(0.98,1.49)	**1.72(1.40,2.08)**
Human development		**1.33(1.13,1.57)**	**0.83(0.75,0.92)**	0.81(0.66,1.15)
**Community controls**				
*Community education*				
Low			1	1
Medium			**3.87(3.64,4.09)**	**2.45(2.30,2.67)**
High			**10.29(9.62,11.01)**	**3.88(3.56,4.23)**
**Community distance problem**				
Low			1	1
High			**0.48(0.46,0.51)**	**0.81(0.78,0.84)**
**Community autonomy**				
Low			1	1
High			**1.22(1.15,1.29)**	**1.10(1.07,1.13)**
**Residence**				
Urban			1	1
Rural			**0.28(0.26,0.30)**	**0.50(0.47,0.53)**
**Individual controls**				
Educational status				
No education				1
Primary				**1.46(1.41,1.56)**
Secondary/higher				**2.75(2.63,2.87)**
**Distance**				
Less problems				1
More problems				**0.81(0.79,0.84)**
**Autonomy**				
No				1
Yes				**1.10(1.07,1.13)**
**Random effects**				
**Country-level**				
Variance (SE)	1.93(0.49)	1.41(0.37)	1.05(0.28)	1.22(0.33)
VPC (%)	22.68	17.49	17.86	20.78
MOR	3.76	3.1	2.66	2.87
**Community-level**				
Variance (SE)	3.29(0.05)	3.36(0.06)	1.54(0.03)	1.36(0.03)
VPC (%)	38.66	41.69	26.19	23.17
MOR	5.64	5.75	3.27	3.04
DIC	210,315.11	198,551.46	196,751.45	163,466.40

Education and voice and accountability are significantly associated with institutional delivery only in Model 2 but loses significance when control variables at both community and individual levels are introduced. Human development index was found to be significantly and positively associated with institutional delivery. However, when individual level variables were introduced in Model 3, the relationship became negative. In Model 4 where all variables are included, the relationship is no longer significant.

Community autonomy is found to be significant here in the sense that women who live in communities in which women have higher decision-making autonomy have higher odds of delivering in institutions compared to those who don’t. It is a logical finding because it is expected that women who are autonomous have a bigger say in ways that resources are distributed within the household. Community education is also an important predictor of institutional delivery. Women who live in communities with more women who are educated up to primary and secondary or higher have better odds of delivering in health facilities than those who do not. At the individual level, there are educational status, female autonomy, and distance to health facilities. All of them were found to be significantly associated with institutional delivery.

Just like in the case of antenatal care visits, the VPC and MOR were calculated to estimate the relative magnitude of variation explained by country and community-level factors and it was established that the combined explanatory share of these high-level factors was larger than individual level factors. The results show that 22.68% and 38.66% of cross-national variation in institutional delivery is accounted for by country and community-level factors respectively. These values remain higher throughout the modelling process even after community and individual level variables are introduced. The MOR also shows values that are considerably larger than 1 indicating the importance of country and community level factors in explaining cross-national variations in institutional delivery.

Civil liberties at the country-level and community level variables remain significant predictors of facility delivery. It is interesting that country-level secondary school female enrolment is no longer a significant predictor when the outcome variable is institutional delivery. The DIC still indicates that the full model, containing individual, community and country-level variables is a better predictor of institutional delivery compared to other models.

### 3.4 Freedom status, capability functionings and postnatal care in SSA

Table 4 reports the results of models explaining postnatal care in sub-Saharan Africa. Country freedom status posits the highest explanatory power on postnatal care in comparison with other outcome variables, and significant relationships are produced in all the models. The results indicate that women living in countries with higher freedom status scores are more likely to receive postnatal check-ups for them and their newly born babies. A one standard deviation increase in country freedom score, increases the odds of postnatal care by a factor of 1.66–2.48.

**Table 4:** Posterior odds ratios for multilevel logistic regression for freedom status, capability functionings and postnatal care in sub-Saharan Africa with 95% credible intervals (N = 245955).

Variable	Model 1	Model 2	Model 3	Model 4
**Country-level factors**				
Intercept	0.82(0.74,1.04)	**0.70(0.60,0.81)**	**0.48(0.42,0.54)**	**0.41(0.32,0.57)**
Freedom status		**1.33(1.10,1.52)**	**1.72(1.35,2.00)**	**1.89(1.66,2.48)**
Female secondary school enrolment		**1.23(1.00,1.34)**	**1.20(1.03,1.43)**	**1.41(1.28,1.53)**
Voice and accountability		0.86(0.70,1.00)	0.88(0.69,1.11)	**1.25(1.11,1.51)**
Human development		0.78(0.68,1.00)	**0.66(0.58,0.78)**	**0.80(0.67,0.98)**
**Community controls**				
**Community education**				
Low			1	1
Medium			**1.79(1.69,1.92)**	**1.45(1.35,1.58)**
High			**2.59(2.45,2.80)**	**1.77(1.60,1.92)**
**Community distance problem**				
Less problems			1	1
More problems			**0.74(0.70,0.78)**	**0.81(0.77,0.86)**
**Community autonomy**				
Low			1	1
High			**1.32(1.25,1.38)**	**1.13(1.10,1.16)**
**Residence**				
Urban			1	1
Rural			**0.84(0.79,0.90)**	1.03(0.93,1.08)
**Individual controls**				
**Educational status**				
No education				1
Primary				**1.24(1.19,1.28)**
Secondary/higher				**1.48(1.41,1.54)**
**Distance**				
Less problems				1
More problems				**0.86(0.83,0.88)**
**Autonomy**				
No				1
Yes				**1.13(1.10,1.16)**
**Random effects**				
Country-level				
Variance (SE)	1.87(0.50)	1.58(0.44)	1.29(0.36)	1.31(0.39)
VPC (%)	27.4	24.12	21.22	21.58
MOR	3.69	3.32	2.95	2.98
Community-level				
Variance (SE)	1.67(0.03)	1.68(0.03)	1.50(0.03)	1.47(0.03)
VPC (%)	24.45	25.65	24.67	24.22
MOR	3.43	3.44	3.22	1.18
DIC	205,651.72	189,817.10	189,523.38	174,088.32

Secondary school female enrolment was not significantly associated with institutional delivery but has a significantly positive influence on postnatal care. Women living in countries with higher secondary school enrolment have a higher propensity of receiving postnatal care. In this cases, a one standard deviation increase in secondary school female employment increases the odds of postnatal care by a factor of 1.28–1.52. School enrolment is consistent across the three models. Surprisingly, human development index is negatively associated with postnatal care across the three models.

Voice and accountability is associated with postnatal care when all other variables are controlled for. It shows that women who live in countries where people have freedom of speech and higher governmental accountability are more likely to access postnatal care. This finding is rather strange because it is usually expected that a predictor variable would be significant before other variables are introduced and not the other way around.

Community autonomy and educational status are other important factors that are found to positively influence postnatal care. Women who live in communities that have more women with decision making authority and in communities with more educated women are more likely to receive postnatal care compared to those who don’t. Distance to health facilities and place or residence at the community level were found to be associated with postnatal care just like in other models. Distance to health facilities, female autonomy and educational status measured at the individual level are also significant predictors of postnatal care.

Just like in other models, the relative importance of factors at the three levels were measured using VPCs and MORs. For postnatal care, cross-national variations are attributable to higher-level factors (community and country-levels) compared to individual-level factors. The VPC for country level factors is 27.4% which means that cross-national variation in postnatal care is significantly attributable to country-level factors while that of community-level factors is 24.45%. The combined contextual level factors explain more than 50% of variations in postnatal care. These values underscore the importance of contextual factors in maternal healthcare utilization.

## 4 Discussion

This study addressed the integration of rights-based and capability approaches in the analysis of maternal healthcare in SSA using freedom status, voice and accountability, female secondary school enrolment, and human development index as well as related controlling variables at the individual and community levels. Freedom status and voice and accountability are important part of the guiding framework of the rights-based approach to health and healthcare. The goal of the rights-based approach to health is to support and sustain good outcomes by analysing and addressing the inequalities, discriminatory practices, and unjust relations in line with the UDHR and other international human rights treaties, which are often at the heart of health problems.

Consistent relationships were found between country-level freedom status and all indicators of maternal healthcare utilization, suggesting that countries which guarantee civil and political liberties to citizen are more likely to also have higher utilization of maternal healthcare service and thus, increasing chances of reducing maternal mortality. The relationship between civil liberties and maternal healthcare utilization is straightforward. The success in implementing human rights obligations, including healthcare depends on the state’s willingness to build a health system based on the human rights approach. Accountability, as articulated by London and Schneider [10] is an important element in the state’s disposition to prioritise human rights obligations. Civil and political liberties encourage strong parliamentary oversight on the executive branch of government in a manner that supports the poor and underprivileged in society and in ways that increases leverage for the health and healthcare sectors. Civil liberties also support strong civil society mobilisation and reinforcing community agency to advance health rights to poor communities [10–12].

Secondary school female enrolment was equally consistently associated with indicators of maternal healthcare utilization, except in the last model for institutional delivery. Education is an important part of the socioeconomic entitlements as well as the capability approach. This study particularly used female secondary school enrolment, and it is plausible that it would positively influence maternal healthcare utilization. Women with secondary education are not only expected to understand the risks associated with failure to use maternal healthcare services but also are more likely to have the resources and decision-making authority essential to access and utilize maternal healthcare in SSA [44–46]. Additionally, secondary education improves chances of women to be employed in formal sector and to have decent income which in turn would influence good maternal health outcomes [47]. In other words, secondary education offers women the capability functionings to circumvent the conditions of vulnerability and claim their rights to maternal healthcare services. This finding is consisted with previous studies which found female education to be an important predictor of maternal and child mortality [48,49].

Community agency is essential in both the rights-based and capability approaches to maternal healthcare utilization because on one hand, it enables women in the community to fight elements of sub-judication by the cultural and social systems, and on the other hand, it gives women and groups the freedom and capabilities to make choices about their own health and healthcare [17]. In this study, community autonomy was analysed, and it was found that it was significantly associated with use of maternal healthcare utilization. This finding gives credibility to the integration of rights-based and capability approaches because, even though they are distinct, they have often times been regarded as complementary [14,17]. Stephen Marks, for example, sees capabilities as starting points of the human rights approach [50].

The relative importance of contextual factors (community and country-level factors) is demonstrated by the between community and country variations in maternal healthcare utilization. For instance, variations in institutional delivery and postnatal care were attributable to community and country-level factors more than individual-level factors. This is the reason why it is important for researchers studying health and healthcare in sub-Saharan Africa to focus more on the influence of broader “upstream” factors as much as they do on individual-level factors [51–53]. High variance partition coefficient values at the community level imply clustering, which suggests that people living in the same community have similar characteristics. This is possible because people living in the same neighbourhoods share culture including economic activities, educational as well as health facilities. The same applies to people of the same country. Although there may be enormous within-country variations, there are some characteristics that are inherent to them by virtue of belonging to the same country. The respect for human rights and government accountability, for example, would affect everyone in the country and would induce variations in certain outcomes like maternal healthcare utilization in comparison with other countries.

The strength of this paper lies in the fact that it is among the first such papers to integrate rights-based approaches with capability approaches to study maternal healthcare in sub-Saharan Africa. It provides evidence of the influence of civil liberties, political rights as well other proximate factors on maternal healthcare utilization. The paper generally adds to a growing number of empirical studies which have shown the importance of rights-based approach in enhancing maternal healthcare utilization as well as maternal and child mortality [48,49]. The use of three-level models provides variance partition values at each level, which gives more information about relative influence of selected independent variables on maternal healthcare utilization. This is important for targeted policy interventions to improve maternal health in sub-Saharan Africa. Additionally, the application of a Bayesian multilevel logistic regression approach addresses the methodological challenges that would be problematic if a standard frequentist approach was used. This is because frequentist inference relies on the assumption of repeated sampling with replacement, which is not the case in the third level unit (country-level) comprising non-stochastic country data [41,42].

The limitations of this study are not different from any study of this nature. Being cross-sectional in design, it uses regression methods which provide only relationships and associations between variables and not causality. However, cross-sectional data may be the best option in low resource countries like SSA, where there is a problem of limited data infrastructure. Another data concern would be the country-level measurements like freedom status, which are constructed with the help of several analysts from different countries. Although every effort has been made to properly calibrate measurements from different sources, it is impossible to rule out biases and prejudices that people may have which may affect their judgements. The findings in this study must be considered in the light of this limitation.

Recall bias may equally be another weakness that is often discussed in analyses which use data collected through survey methods. Recall bias is taken to mean the likely failure of research respondents to recall information properly due to the time lapse between relevant events and the interview. This may be true for this study about the DHS data [54]. However, it should be noted that the study focused on life-changing events of pregnancy and childbirth and therefore the possibility of forgetting when such matters are involved is minimal. Moreover, the DHS is conducted with enormous rigour by well-trained personnel.

In terms of policy implications, this study makes a few recommendations. Having found that civil and political liberties are significantly associated with maternal healthcare utilization, we recommend that the rights-based and capability approaches be considered integral in public health policy strategies aimed at bolstering use of maternal health services in sub-Saharan Africa. In this regard, it is important for governments to support mechanisms that promote freedoms at the country-level. We also recommend that national governments in sub-Saharan Africa begin to prioritize higher female secondary school enrolments as a matter of public policy directives. Female secondary education gives women better head starts in life compared to basic education which has always been the priority of national governments in sub-Saharan Africa.

## 5 Conclusion

This study integrated the rights-based and capabilities approach to analyse maternal healthcare utilization in sub-Saharan Africa. Freedom status and voice and accountability were regarded as proxies for civil and political liberties, which are the fulcrum of the rights-based approach. The female secondary school enrolment variable is a proxy for the capability functionings at the country level. Related individual and community level factors were controlled for. The results indicated that generally, freedoms and capability functionings are positively associated with maternal healthcare utilization in sub-Saharan Africa. The explanation for these associations may be that freedom opens room for strong people, parliamentary and civil society oversight holding governments accountable, which results in better provision of social, health and economic services to the larger population. Therefore, improving civil liberties and political rights in a country, coupled with enhanced women’s autonomy and access to education at the individual and community levels, can bolster maternal healthcare utilization in sub-Saharan Africa. However, most of the variables at the country-level are composite, which makes it difficult to establish specific dimensions of the composite variable that have contributed to maternal healthcare utilization. It is important for further research to determine specific causal pathways between human rights and maternal healthcare utilization. Further research could also examine the extent to which the rights-based approach influences maternal healthcare, relative to other factors such as gender norms, health system characteristics, health insurance, resource allocation and patient behaviour among other factors.
